# Genetics of Non-Alcoholic Fatty Liver and Cardiovascular Disease: Implications for Therapy?

**DOI:** 10.3389/fphar.2019.01413

**Published:** 2020-01-08

**Authors:** Karthik Chandrasekharan, William Alazawi

**Affiliations:** Barts Liver Centre, Blizard Institute, Queen Mary University of London, London, United Kingdom

**Keywords:** non-alcoholic fatty liver disease, non-alcoholic steatohepatitis, cardiovascular disease, genome wide association studies, therapeutics

## Abstract

Non-alcoholic fatty liver disease (NAFLD) is the most common cause of chronic liver disease worldwide. The most common cause of mortality in NAFLD is cardiovascular disease (CVD), and a key of focus in drug development is to discover therapies that target both liver injury and CVD risk. NAFLD and CVD are complex disease spectra with complex heritability patterns. Nevertheless, genome wide association studies and meta-analyses of these have identified genetic loci that are associated with increased risk of relevant pathological features of disease or clinical endpoints. This review focuses on the genetic risk loci identified in the NAFLD spectrum and asks whether any of these are also risk factors for CVD. Surprisingly, given the shared co-morbidities and risk factors, little robust evidence exists that NAFLD and CVD share genetic risk. Despite this, therapeutic intervention that targets both liver disease and CVD remains an important clinical need and a major focus for pharmaceutical development.

## Body

Non-alcoholic fatty liver disease (NAFLD) affects over 20% of people in the West, and up to one third of people living in South America or the Middle East (Younossi, 2019). NAFLD is defined by the presence of hepatic steatosis in the absence of a secondary cause (e.g., excessive alcohol consumption or drugs). It is associated with hypertension and a number of metabolic co-morbidities, including obesity, type 2 diabetes mellitus (T2DM), hyperlipidemia, and metabolic syndrome (Younossi et al., 2016). NAFLD is a disease spectrum that comprises simple steatosis, non-alcoholic steatohepatitis (NASH), and fibrosis which can lead to cirrhosis and hepatocellular carcinoma (HCC) (Chalasani et al., 2012; White et al., 2012; Alexander et al., 2019b). The determinants of progression through this spectrum are not completely understood, but increased visceral adiposity, insulin resistance, lipotoxicity, inflammation, environmental factors such as diet as well as genetics are likely to play a role. There are currently no drugs licensed for the treatment of the NAFLD spectrum although many are in development.

A large body of evidence indicates that cardiovascular disease (CVD) is a common cause of death among patients with NAFLD (Rafiq et al., 2009; Söderberg et al., 2010; Stepanova and Younossi, 2012; Angulo et al., 2015; Targher et al., 2016; Mahfood Haddad et al., 2017; Younossi et al., 2017) although this is not a universal finding (Lazo et al., 2011; Wu et al., 2016; Zeb et al., 2016; Alexander et al., 2019a; Morrison et al., 2019). Some of this dissonance may relate to variable adjustment for existing risk factors including diabetes, obesity, smoking, ethnicity, and social deprivation. Therefore, while some evidence suggests increased CVD risk, and the two disease states share common pathological mechanisms [e.g., oxidative stress, inflammation, and endothelial dysfunction (Liu and Lu, 2014; Francque et al., 2016)], a causal relationship has yet to be proven.

Nevertheless, there is a drive to discover therapeutic strategies that combine hepatic efficacy with reducing CVD risk in this multi-morbid population who carry many metabolic risk factors. One possible solution may lie in the genetics of these conditions. If NASH and CVD are closely linked, it is plausible to hypothesize that genetic variants that confer adverse risk and that are shared between disease states encode potential therapeutic targets that may meet treatment goals. Over 163 genetic loci are associated with CVD (Erdmann et al., 2018), and a much smaller number of loci with NAFLD spectrum (Eslam et al., 2018) but there is minimal overlap between these groups of genes. Here, we review the genetic associations with disease states in the NAFLD spectrum and the evidence, if any, of their role in CVD.

### Genetics of Non-Alcoholic Fatty Liver Disease

Genome wide association studies (GWAS) have enabled the association of genetic polymorphisms with disease state or treatment response (Eslam et al., 2018). Limitations of GWAS include absence of causality, requirement for very large sample sizes and difficulties in interpreting heritability in complex polygenic conditions such as NAFLD (Tam et al., 2019). Different endpoints have been used in NAFLD GWAS including histology, imaging-based hepatic fat content, serum liver enzymes, and presence of HCC, contributing to heterogeneity. Here we focus primarily on candidate NAFLD risk genes with consistent results in discovery GWAS or meta-analyses of GWAS, and validated using candidate gene studies, a strategy described in a recent study clustering NAFLD associated genes (Brouwers et al., 2019). We also highlight other genes not identified with GWAS, but have significant associations with CVD in retrospective studies or meta-analyses of retrospective studies, and outline their association with NAFLD (**Table 1**).

**Table 1:** Summary of genetic variants and their effects on non-alcoholic fatty liver disease/non-alcoholic steatohepatitis.

Genetic variant	Effect	Reference(s)
PNPLA3 I148M	Increases hepatic fat content, and risk of hepatic steatosis, fibrosis, and HCC	(Romeo et al., 2008; Sookoian et al., 2009; Liu et al., 2014a)
TM6SF2 E167K	Increases levels of hepatic triglyceride content, ALT, and increased risk of hepatic fibrosis	(Kozlitina et al., 2014; Liu et al., 2014b)
MBOAT7 rs614738	Increases hepatic triglyceride content, and risk of hepatic steatosis, fibrosis, and HCC	(Buch et al., 2015; Mancina et al., 2016; Donati et al., 2017)
GCKR rs780094	Increased hepatic steatosis and fibrosis	(Speliotes et al., 2011; Petta et al., 2014)
NCAN P92S	Increased risk of hepatic steatosis, lobular inflammation, and fibrosis	(Speliotes et al., 2011; Gorden et al., 2013)
PPP1R3B rs4240624	Increased risk of hepatic steatosis	(Speliotes et al., 2011)
PPP1R3B rs6175625	Predictor of ultrasonography diagnosed NAFLD	(Di Costanzo et al., 2018)
TRIB1 rs2954021	Associated with raised ALT and increased risk of histological or ultrasonography diagnosed NAFLD	(Chambers et al., 2011; Kitamoto et al., 2014; Liu et al., 2019)
ERLIN1-CHUK-CWF19L1	Increased risk of CT-diagnosed NAFLD and raised ALT	(Feitosa et al., 2013)
PEMT V175M	Increased risk of histologically diagnosed NAFLD and NASH, and ultrasonography/MRS diagnosed NAFLD	(Tan et al., 2016)
MTTP 493G > T	Increased risk of biopsy proven NAFLD and NASH, and ultrasonography diagnosed NAFLD	(Zheng et al., 2014)
SOD2 rs4880	Increased risk of fibrosis steatohepatitis and fibrosis	(Al-Serri et al., 2012)
UCP2 866 G > A	Reduced risk of NASH, more pronounced effect in those without impaired fasting glucose or diabetes mellitus	(Fares et al., 2015)

### Patatin-Like Phospholipase Domain-Containing Protein 3 (Aliases: Adiponutrin, Calcium-Independent Phospholipase A2-Epsilon, or Acylglycerol O-Acyltransferase)

PNPLA3 hydrolyses triglycerides and retinyl esters (Huang et al., 2011) and is associated with NAFLD in GWAS (Romeo et al., 2008). The I148M variant (isoleucine to methionine substitution at amino acid position 148) of *PNPLA3* has reduced hydrolase activity and impairs retinyl ester release, resulting in accumulation of triglycerides and retinyl esters within hepatocytes and stellate cells and increased hepatic fat content (Pingitore et al., 2014; Pirazzi et al., 2014; BasuRay et al., 2017). Upregulation of wild-type *PNPLA3* reduces secretion of matrix metalloproteinases and tissue inhibitor of metalloproteinases, protecting against fibrosis (Pingitore et al., 2016).

*PNPLA3* I148M (rs738409 C > G) is significantly associated with imaging-assessed hepatic fat content, adjusted for body mass index (BMI), diabetes status, ethanol use, and ethnicity (p = 7.0x10^−14^) (Romeo et al., 2008). This allele significantly increases the risk of hepatic steatosis and fibrosis independent of age, sex, BMI, and homeostatic model of insulin resistance (HOMA) index, in a “dose-dependent” manner, with intermediate severity of steatosis in heterozygotes and more severe steatosis in homozygotes (G/G) (Sookoian et al., 2009; Valenti et al., 2010). Cross-sectional studies show the G allele is more frequent in histologically confirmed steatosis (frequency: 49.2%, p = 2x10^−3^) and NASH (frequency: 51.8%, p = 4x10^−26^) compared with normal controls (frequency: 22.8%). Risk of bridging fibrosis increases with each G allele (OR 1.50, 95% CI 1.19–1.89), independently of steatosis and inflammation (Rotman et al., 2010). The rs738409 C > G polymorphism is also associated with increased HCC risk (OR: 2.046, 95% CI 1.47–2.84, χ^2^^=^ 18.50, p < 0.0001), and additive modeling showed that G/G homozygosity conferred a fivefold increase in risk compared with C/C homozygous NAFLD controls (OR: 5.05, 95% CI 1.47–17.29, p = 0.01) (Liu et al., 2014a). A meta-analysis of 24 studies with a total of 9,195, mostly Caucasian, patients concluded that PNPLA3 I148M is significantly associated with increased risk of advanced fibrosis in individuals with NAFLD (OR: 1.23, 95% CI 1.10–1.37) and of HCC (OR: 1.67, 95% CI 1.27–2.21) in patients with NAFLD (Singal et al., 2014).

In non-Caucasian cohorts, there is also strong evidence for an association of PNPLA3 with NAFLD. In two Japanese cohorts the PNPLA3 rs738409 variant was significantly associated with NAFLD (OR: 1.66, 95% CI 1.43–1.94, p = 1.4x10^−10^), and, in particular, a more severe histological subtype (Kawaguchi et al., 2012; Kitamoto et al., 2013), with similar findings in a large Korean cohort (Chung et al., 2018). PNPLA3 I148M is therefore strongly associated with the NAFLD spectrum in both Caucasian and non-Caucasian cohorts.

### Transmembrane 6 Superfamily 2

TM6SF2 encodes a protein that localizes to the endoplasmic reticulum (ER)-Golgi apparatus of hepatocytes, and increases secretion of triglyceride-rich lipoproteins; reduced TM6SF2 expression increases hepatic triglyceride content (Mahdessian et al., 2014). TM6SF2 E167K variant (rs58542926 C > T) is associated with increased levels of hepatic triglyceride content measured using ^1^H-MRS (independently of PNPLA3 I148M), raised serum alanine transaminase (ALT), and reduced serum cholesterol and triglycerides, in a multi-ethnic cohort (Kozlitina et al., 2014). The association with serum ALT was confirmed in two further cohorts, of 8,585 European Americans and 73,532 individuals from two Copenhagen-based studies (Kozlitina et al., 2014). This variant is associated with increased hepatic fibrosis (β: 0.549 ± 0.135, 95% CI 0.285–1.813, p = 5.57x10^−5^), again independently of PNPLA3 I148M (Liu et al., 2014b). After adjusting for confounding variables, the co-existence of PNPLA3 rs738409 and TM6SF2 rs58542926 in a Korean cohort increased the risk of NASH (OR per risk allele: 2.03, 95% CI 1.50–2.73 p < 0.001) and significant fibrosis (OR for each risk allele: 1.61, 95% CI 1.19–2.17, p < 0.002) (Koo et al., 2018) with similar findings from a Chinese cohort (OR for each allele 1.52, regression line R^2^ = 0.992) (Wang et al., 2016). Thus TM6SF2 E167K is associated with stages of NAFLD independently of PNPLA3 I148M.

### Membrane Bound O-Acyltransferase Domain Containing 7

MBOAT7 is highly expressed in hepatocytes, hepatic stellate cells, and hepatic sinusoidal cells, and the rs641738 T allele increases risk of hepatic triglyceride content and NAFLD spectrum, with each T allele increasing the risk of steatosis (OR: 1.42, 95% CI 1.07–1.91, p = 0.015), NASH (OR: 1.18, 95% CI 1.00–1.40, p = 0.05), hepatic fibrosis stage F2-4 (OR: 1.30, 95% CI 1.06–1.70, p = 0.012), and HCC in the absence of advanced fibrosis (OR: 2.10, 95% CI 1.33–3.31) (Buch et al., 2015; Mancina et al., 2016; Donati et al., 2017). *MBOAT7, PNPLA3*, and *TM6SF2* risk loci confer a stepwise risk of increased hepatic fat content per each additional risk allele (Mancina et al., 2016).

### Glucokinase Regulatory Protein

The GCKR rs780094 polymorphism is significantly associated with CT- and histologically proven hepatic steatosis in GWAS (OR = 1.45, p = 2.59x10^−8^) (Speliotes et al., 2011) and in meta-analysis of five studies (2,091 NAFLD cases and 3,003 controls) (OR: 1.25, 95% CI 1.14–1.36, p < 0.00001, I^2^ = 0%) (Zain et al., 2015). This polymorphism is associated with higher expression of GCKR (Rodríguez et al., 2018), and with reduced risk of T2DM (Sparsø et al., 2008; Onuma et al., 2010). In patients with NAFLD the rs780094 C > T polymorphism is significantly associated with hepatic fibrosis stage > F1 (OR: 2.06, 95% CI 1.02–1.14, p = 0.0008) (Petta et al., 2014). The rs1260326 P446L variant is not inhibited by fructose-6-phosphate, resulting in increased activity of glucokinase, hepatic uptake of glucose (Beer et al., 2009), and *de novo* lipogenesis (Anstee and Day, 2013). In individuals who carried GCKR rs1260326, *PNPLA3* rs738409, *TM6SF2* rs58542926, and *MBOAT7* rs641738 variants (n = 218 NAFLD/445 total) there was a fivefold increased risk of steatosis (OR: 4.97, 95% CI 2.51–9.83) (Di Costanzo et al., 2018).

### Neurocan Core Protein

The Speliotes GWAS identified a number of other loci associated with NAFLD. NCAN rs2228603 T variant (P92S) is associated with increased risk of histological steatosis (1.22, SE 0.07 in presence of T allele *vs.* 1.03, SE 0.03 for CC genotype, p = 0.03), lobular inflammation (39.7% in presence of T allele *vs.* 30.1% for CC genotype, p = 0.02), and presence of fibrosis (27.8% in presence of T allele *vs.* 17.2% for CC genotype, p = 0.002) in patients undergoing bariatric surgery (Gorden et al., 2013). NCAN is a proteoglycan involved in remodeling central nervous system extracellular matrix (Rauch et al., 2001), also expressed in the liver (Nischalke et al., 2014).

### Protein Phosphatase 1 Regulatory Subunit 3B

PPP1R3B rs4240624 is associated with CT-diagnosed hepatic steatosis, but not histological evidence of inflammation and fibrosis (Speliotes et al., 2011) and the *PPP1R3B* rs61756425 variant is a strong predictor of severe NAFLD on ultrasound (OR: 32.6, 95% CI 4.22–251.4, p = 0.001) (Di Costanzo et al., 2018). The mutated PPP1R3B results in excess hepatic glycogen (but not triglyceride), resulting in hepatic injury (Stender et al., 2018).

### Tribbles Pseudokinase 1

TRIB1 rs2954021 G > A allele is significantly associated with raised ALT (Chambers et al., 2011) and histologically or ultrasound-diagnosed NAFLD (OR: 1.52, 95% CI 1.23–1.88, p = 9.7x10^−5^) (OR: 2.050, 95% CI 1.110–3.786, p = 0.022) (Kitamoto et al., 2014; Liu et al., 2019).

### Genetic Associations Not Discovered Through Genome Wide Association Studies

A correlated meta-analysis identified nine *ERLIN1-CHUK-CWF19L1* gene variants associated with CT-diagnosed NAFLD and raised ALT levels (Feitosa et al., 2013). *Phosphatidylethanolamine N-methyltransferase (PEMT)* loss of function results in impaired lipid removal from the liver due to reduced levels of hepatic phosphatidylcholine, which mediates triglyceride secretion from hepatocytes (Song et al., 2005; Vance, 2013). Although not demonstrated in GWAS, a meta-analysis of 6 studies showed that the PEMT rs7046 A allele (V175M) is associated with NAFLD in East-Asian populations (OR 1.55, 95% CI 1.13–2.11, p = 0.005) (Tan et al., 2016). *Mitochondrial transport protein (MTTP)* 493 G > T polymorphism is associated with NAFLD (G *vs.* T allele OR: 1.46, 95% CI 1.20–1.78, p < 0.001), albeit in meta-analysis, not GWAS (Zheng et al., 2014). *Superoxide dismutase 2 (SOD2)* is involved in removal of reactive oxygen species in mitochondria (Wispe’ et al., 1989). In a case-control study of 502 patients with biopsy-proven NAFLD, SOD2 rs4880 was associated with a dose-related increased risk of advanced fibrosis (p = 0.008 for trend) but not steatohepatitis (Al-Serri et al., 2012). *Uncoupling protein 2 (UCP2)* has a role in regulating lipid efflux and oxidative metabolism, and homozygous 866 G > A is associated with increased hepatic protein levels and reduced risk of NASH (adjusted OR: 0.49, 95% CI: 0.26–0.90, p = 0.02) (Fares et al., 2015).

### Are There Common Genetic Associations Shared Between Non-Alcoholic Fatty Liver Disease and Cardiovascular Disease?

Cardiovascular disease is a broad term comprising coronary artery disease (CAD), cerebrovascular disease, and peripheral arterial disease that are largely driven by atherosclerosis (Stewart et al., 2017). While events such as fatal or non-fatal myocardial infarction or stroke may be clinically more meaningful, surrogate markers of CVD including carotid intima-media thickness, carotid artery plaques, and CT-based coronary artery calcification have been used as study endpoints (Patil and Sood, 2017). The genetics of CVD that have identified over 160 loci associated with CAD at a genome-wide level of significance have been reviewed elsewhere (Erdmann et al., 2018).

*PNPLA3* I148M allele had a small, statistically significant (albeit not to GWAS levels of significance) protective effect (OR: 0.92, 95% CI 0.87–0.97, p = 0.002) against CVD, in a meta-analysis of 48 GWAS including 60,801 cases with CVD (Simons et al., 2017). This may relate to reduced levels of circulating triglycerides (Tang et al., 2015). A recent prospective study of patients undergoing elective coronary angiography found that while there was a non-significant trend for a protective effect of the presence of PNPLA3 I148M and CAD >75% stenosis, when corrected for age, sex, use of statins, and serum high-density lipoprotein, there was a significant protective effect (OR: 0.21, 95% CI 0.05–0.88, p = 0.03) (Rüschenbaum et al., 2018). Mendelian randomization has been used to assess the association of *PNPLA3* I148M with ischemic heart disease (IHD), and although the risk of IHD increases with increased hepatic fat content, for which *PNPLA3* I148M is a risk factor, there was no association with IHD (OR per M allele = 0.98, 95% CI 0.95–1.02, p = 0.79) (Lauridsen et al., 2018).

Interestingly, presence of PNPLA3 I148M variant has been shown to confer a small, but significant risk of premature CAD (defined as MI, angioplasty, revascularization surgery, coronary stenosis >50% diagnoses before the age of 55 in men, and 65 in women) only in those with T2DM (OR 1.20, 95% CI 1.01–1.42, p = 0.042), in a study of 1,103 individuals with premature CAD and 1,469 healthy controls (Posadas-Sánchez et al., 2017). Homozygous PNPLA3 I148M genotype confers greater risk of more severe carotid artery intima-media thickness (IMT) in 162 patients with biopsy-proven NAFLD aged < 50 (OR 2.94, 95% CI 1.12–7.70, p = 0.02), validated in 267 patients with clinical or histological NAFLD (Petta et al., 2013).

The *TM6SF2 E167K* variant which is associated with hepatic steatosis, fibrosis, and HCC development was protective against CVD with lower total cholesterol, LDL-cholesterol, and serum triglycerides in a meta-analysis of 10 studies (Pirola and Sookoian, 2015). A further meta-analysis of 48 GWAS including 60,801 cases with CVD confirmed a small reduction in CVD risk with *TM6SF2* E167K (OR: 0.95, 95% CI 0.92–0.98, p = 0.005) (Simons et al., 2017), and in a smaller cross-sectional study, it was associated with reduced risk of carotid artery plaques (OR: 0.49, 95% CI 0.25–0.94) (Dongiovanni et al., 2015). As described, this variant is associated with raised serum ALT levels, however a recent Mendelian randomization study has also suggested that raised ALT levels may reduce the risk of IHD, and this may be due to a reduction in serum triglyceride levels (Xu et al., 2017).

*PEMT* rs12936587 variant was associated with increased susceptibility to CAD (OR 1.07, 95% CI 1.05–1.09, p = 4.45x10^−10^) (Schunkert et al., 2011) in a meta-analysis of 14 GWAS (22,233 cases of CAD and 64,762 controls). However, there was no association with cardiovascular events (IHD, CVA, peripheral arterial disease), and atherosclerosis determined by carotid intima-media wall thickness (IMT) by carotid ultrasonography in a detailed study of 2,609 Spanish individuals (López-Mejías et al., 2017).

*TRIB1* rs2954021 variant is associated with increased risk of IHD (15% increased risk in AA *vs.* TT genotype, 95% CI 5–20%) and MI (17% increased risk in AA *vs.* TT genotype, 95% CI 6–30%) (Varbo Anette et al., 2011) (CARDIoGRAMplusC4D Consortium et al., 2013). TRIB1 rs2954021 and rs231150 are associated with increased risk of coronary heart disease (rs2954021 OR for A *vs.* T allele in log-additive model: 1.36, 95% CI 1.14–1.63, p < 0.001, rs231150 OR for A *vs.* T allele in log-additive model: 1.36, 95% CI 1.14–1.63, p < 0.0015), and TRIB 1 rs2954021 is also associated with increased risk of ischemic stroke across genetic models (OR for A *vs.* T allele in log-additive model: 1.30, 95% CI 1.09–1.55, p = 0.0039) (Zhang et al., 2019). However, in this study controls were not matched for key risk factors and patients with CAD and stroke had higher BMI, systolic blood pressure, pulse pressure, and triglyceride levels and lower prevalence of alcohol consumption, and ratio of ApoA1:ApoB.

The clustering of 12 genetic variants associated with NAFLD by weighted fixed-effects statistical modeling showed no association with CAD or MI (OR: 1.00, 95% CI 0.99–1.01, p = 0.93) (Brouwers et al., 2019). Restricting this analysis to the four most validated genes (*PNPLA3*, *TM6SF2*, *GCKR*, and *MBOAT7*) also resulted in a null association (OR: 0.99, 95% CI 0.98–1.00) (Brouwers et al., 2019).

### Discussion and Implications on Treatment of Non-Alcoholic Fatty Liver Disease

GWAS have identified genes that are associated with NAFLD, many of which have been further validated in observational data. Though GWAS do have certain limitations as described, there are at least 4 genes that have been extensively validated in their association with NAFLD. However, the genes most robustly associated with NAFLD do not appear to contribute to increased cardiovascular risk in terms of CAD, and in fact *TM6SF2* E167K is protective against CVD (IHD and ischemic stroke), and *PNPLA3* I148M exerts no significant effect on CVD. Where associations have been described in *TRIB1* and *PEMT* (albeit different SNPs), the studies have shown null associations with ‘hard’ clinical endpoints or suffered incomplete matching of controls. This does not mean that there is no independent association of fatty liver with CVD; rather the complex relationship probably requires much larger studies powered to detect the association as well as Mendelian randomization and interaction studies.

Given that no drugs are licensed to treat the NAFLD spectrum, management focuses on lifestyle modification. Ideally, therapy would treat liver disease, and also improve clinical endpoints including mortality. Therefore improved patient outcomes may be achieved by interventions that improve control of T2DM, lipids, blood pressure, and obesity rather than have profound effects in the liver. While there is evidence to support a degree of genetic risk in each disease spectrum individually, there is little to no evidence of any shared genetic risk. Therefore while knowledge of genetic loci is potentially useful for risk stratification of patients with NAFLD, therapeutic targeting of the products of these genes as a strategy that improves both liver and cardiovascular health has yet to be proven.

## Author Contributions

KC and WA both wrote and edited the manuscript.

## Conflict of Interest

WA has received speaking honoraria and consultancy fees from Gilead, Intercept, GSK, Allergan, and Goldman Sachs.

The remaining author declares that the research was conducted in the absence of any commercial or financial relationships that could be construed as a potential conflict of interest.

## References

[B1] Alexander M. Loomis A. K. Lei J. V. D. Duarte-Salles T. Prieto-Alhambra D. Ansell D. (2019a). Non-alcoholic fatty liver disease and risk of incident acute myocardial infarction and stroke: findings from matched cohort study of 18 million European adults. BMJ 367, l5367. 10.1136/bmj.l5367 31594780 PMC6780322

[B2] Alexander M. Loomis A. K. van der Lei J. Duarte-Salles T. Prieto-Alhambra D. Ansell D. (2019b). Risks and clinical predictors of cirrhosis and hepatocellular carcinoma diagnoses in adults with diagnosed NAFLD: real-world study of 18 million patients in four European cohorts. BMC Med. 17, 95. 10.1186/s12916-019-1321-x 31104631 PMC6526616

[B3] Al-Serri A. Anstee Q. M. Valenti L. Nobili V. Leathart J. B. S. Dongiovanni P. (2012). The SOD2 C47T polymorphism influences NAFLD fibrosis severity: evidence from case-control and intra-familial allele association studies. J. Hepatol. 56, 448–454. 10.1016/j.jhep.2011.05.029 21756849

[B4] Angulo P. Kleiner D. E. Dam-Larsen S. Adams L. A. Bjornsson E. S. Charatcharoenwitthaya P. (2015). Liver fibrosis, but no other histologic features, is associated with long-term outcomes of patients with nonalcoholic fatty liver disease. Gastroenterology 149, 389–397.e10. 10.1053/j.gastro.2015.04.043 25935633 PMC4516664

[B5] Anstee Q. M. Day C. P. (2013). The genetics of NAFLD. Nat. Rev. Gastroenterol. Hepatol. 10, 645–655. 10.1038/nrgastro.2013.182 24061205

[B6] BasuRay S. Smagris E. Cohen J. C. Hobbs H. H. (2017). The PNPLA3 variant associated with fatty liver disease (I148M) accumulates on lipid droplets by evading ubiquitylation. Hepatology 66, 1111–1124. 10.1002/hep.29273 28520213 PMC5605398

[B7] Beer N. L. Tribble N. D. McCulloch L. J. Roos C. Johnson P. R. V. Orho-Melander M. (2009). The P446L variant in GCKR associated with fasting plasma glucose and triglyceride levels exerts its effect through increased glucokinase activity in liver. Hum. Mol. Genet. 18, 4081–4088. 10.1093/hmg/ddp357 19643913 PMC2758140

[B8] Brouwers M. C. G. J. Simons N. Stehouwer C. D. A. Koek G. H. Schaper N. C. Isaacs A. (2019). Relationship between nonalcoholic fatty liver disease susceptibility genes and coronary artery disease. Hepatol. Commun. 3, 587–596. 10.1002/hep4.1319 30976747 PMC6442707

[B9] Buch S. Stickel F. Trépo E. Way M. Herrmann A. Nischalke H. D. (2015). A genome-wide association study confirms *PNPLA3* and identifies *TM6SF2* and *MBOAT7* as risk loci for alcohol-related cirrhosis. Nat. Genet. 47, 1443–1448. 10.1038/ng.3417 26482880

[B10] CARDIoGRAMplusC4D Consortium Deloukas P. Kanoni S. Willenborg C. Farrall M. Assimes T. L. (2013). Large-scale association analysis identifies new risk loci for coronary artery disease. Nat. Genet. 45, 25–33. 10.1038/ng.2480 23202125 PMC3679547

[B11] Chalasani N. Younossi Z. Lavine J. E. Diehl A. M. Brunt E. M. Cusi K. (2012). The diagnosis and management of non-alcoholic fatty liver disease: practice guideline by the American association for the study of liver diseases, American College of Gastroenterology, and the American Gastroenterological Association. Hepatology 55, 2005–2023. 10.1002/hep.25762 22488764

[B12] Chambers J. C. Zhang W. Sehmi J. Li X. Wass M. N. Van der Harst P. (2011). Genome-wide association study identifies loci influencing concentrations of liver enzymes in plasma. Nat. Genet. 43, 1131–1138. 10.1038/ng.970 22001757 PMC3482372

[B13] Chung G. E. Lee Y. Yim J. Y. Choe E. K. Kwak M.-S. Yang J. I. (2018). Genetic Polymorphisms of PNPLA3 and SAMM50 are associated with nonalcoholic fatty liver disease in a Korean Population. Gut Liver 12, 316–323. 10.5009/gnl17306 29271184 PMC5945263

[B14] Di Costanzo A. Belardinilli F. Bailetti D. Sponziello M. D’Erasmo L. Polimeni L. (2018). Evaluation of polygenic determinants of non-alcoholic fatty liver disease (NAFLD) by a candidate genes resequencing strategy. Sci. Rep. 8, 3702. 10.1038/s41598-018-21939-0 29487372 PMC5829219

[B15] Donati B. Dongiovanni P. Romeo S. Meroni M. McCain M. Miele L. (2017). MBOAT7 rs641738 variant and hepatocellular carcinoma in non-cirrhotic individuals. Sci. Rep. 7, 4492. 10.1038/s41598-017-04991-0 28674415 PMC5495751

[B16] Dongiovanni P. Petta S. Maglio C. Fracanzani A. L. Pipitone R. Mozzi E. (2015). Transmembrane 6 superfamily member 2 gene variant disentangles nonalcoholic steatohepatitis from cardiovascular disease. Hepatol. Baltim. 61, 506–514. 10.1002/hep.27490 25251399

[B17] Erdmann J. Kessler T. Munoz Venegas L. Schunkert H. (2018). A decade of genome-wide association studies for coronary artery disease: the challenges ahead. Cardiovasc. Res. 114, 1241–1257. 10.1093/cvr/cvy084 29617720

[B18] Eslam M. Valenti L. Romeo S. (2018). Genetics and epigenetics of NAFLD and NASH: Clinical impact. J. Hepatol. 68, 268–279. 10.1016/j.jhep.2017.09.003 29122391

[B19] Fares R. Petta S. Lombardi R. Grimaudo S. Dongiovanni P. Pipitone R. (2015). The UCP2 -866 G > A promoter region polymorphism is associated with nonalcoholic steatohepatitis. Liver Int. 35, 1574–1580. 10.1111/liv.12707 25351290

[B20] Feitosa M. F. Wojczynski M. K. North K. E. Zhang Q. Province M. A. Carr J. J. (2013). The ERLIN1-CHUK-CWF19L1 gene cluster influences liver fat deposition and hepatic inflammation in the NHLBI Family Heart Study. Atherosclerosis 228, 175–180. 10.1016/j.atherosclerosis.2013.01.038 23477746 PMC3640729

[B21] Francque S. M. Graaff D. V. D. Kwanten W. J. (2016). Non-alcoholic fatty liver disease and cardiovascular risk: pathophysiological mechanisms and implications. J. Hepatol. 65, 425–443. 10.1016/j.jhep.2016.04.005 27091791

[B22] Gorden A. Yang R. Yerges-Armstrong L. M. Ryan K. A. Speliotes E. Borecki I. B. (2013). Genetic variation at NCAN locus is associated with inflammation and fibrosis in non-alcoholic fatty liver disease in morbid obesity. Hum. Hered. 75, 34–43. 10.1159/000346195 23594525 PMC3864002

[B23] Huang Y. Cohen J. C. Hobbs H. H. (2011). Expression and Characterization of a PNPLA3 Protein Isoform (I148M) Associated with nonalcoholic fatty liver disease. J. Biol. Chem. 286, 37085–37093. 10.1074/jbc.M111.290114 21878620 PMC3199456

[B24] Kawaguchi T. Sumida Y. Umemura A. Matsuo K. Takahashi M. Takamura T. (2012). Genetic polymorphisms of the human PNPLA3 gene are strongly associated with severity of non-alcoholic fatty liver disease in Japanese. PloS One 7, e38322. 10.1371/journal.pone.0038322 22719876 PMC3375283

[B25] Kitamoto T. Kitamoto A. Yoneda M. Hyogo H. Ochi H. Nakamura T. (2013). Genome-wide scan revealed that polymorphisms in the PNPLA3, SAMM50, and PARVB genes are associated with development and progression of nonalcoholic fatty liver disease in Japan. Hum. Genet. 132, 783–792. 10.1007/s00439-013-1294-3 23535911

[B26] Kitamoto A. Kitamoto T. Nakamura T. Ogawa Y. Yoneda M. Hyogo H. (2014). Association of polymorphisms in GCKR and TRIB1 with nonalcoholic fatty liver disease and metabolic syndrome traits. Endocr. J. 61, 683–689. 10.1507/endocrj.EJ14-0052 24785259

[B27] Koo B. K. Joo S. K. Kim D. Bae J. M. Park J. H. Kim J. H. (2018). Additive effects of PNPLA3 and TM6SF2 on the histological severity of non-alcoholic fatty liver disease. J. Gastroenterol. Hepatol. 33, 1277–1285. 10.1111/jgh.14056 29193269

[B28] Kozlitina J. Smagris E. Stender S. Nordestgaard B. G. Zhou H. H. Tybjærg-Hansen A. (2014). Exome-wide association study identifies a TM6SF2 variant that confers susceptibility to nonalcoholic fatty liver disease. Nat. Genet. 46, 352–356. 10.1038/ng.2901 24531328 PMC3969786

[B29] López-Mejías R. Corrales A. Vicente E. Robustillo-Villarino M. González-Juanatey C. Llorca J. (2017). Influence of coronary artery disease and subclinical atherosclerosis related polymorphisms on the risk of atherosclerosis in rheumatoid arthritis. Sci. Rep. 7, 40303. 10.1038/srep40303 28059143 PMC5216400

[B30] Lauridsen B. K. Stender S. Kristensen T. S. Kofoed K. F. Køber L. Nordestgaard B. G. (2018). Liver fat content, non-alcoholic fatty liver disease, and ischaemic heart disease: Mendelian randomization and meta-analysis of 279 013 individuals. Eur. Heart J. 39, 385–393. 10.1093/eurheartj/ehx662 29228164

[B31] Lazo M. Hernaez R. Bonekamp S. Kamel I. R. Brancati F. L. Guallar E. (2011). Non-alcoholic fatty liver disease and mortality among US adults: prospective cohort study. BMJ 343, d6891. 10.1136/bmj.d6891 22102439 PMC3220620

[B32] Liu H. Lu H.-Y. (2014). Nonalcoholic fatty liver disease and cardiovascular disease. World J. Gastroenterol. 20, 8407–8415. 10.3748/wjg.v20.i26.8407 25024598 PMC4093693

[B33] Liu Y.-L. Patman G. L. Leathart J. B. S. Piguet A.-C. Burt A. D. Dufour J.-F. (2014a). Carriage of the PNPLA3 rs738409 C > G polymorphism confers an increased risk of non-alcoholic fatty liver disease associated hepatocellular carcinoma. J. Hepatol. 61, 75–81. 10.1016/j.jhep.2014.02.030 24607626

[B34] Liu Y.-L. Reeves H. L. Burt A. D. Tiniakos D. McPherson S. Leathart J. B. S. (2014b). *TM6SF2* rs58542926 influences hepatic fibrosis progression in patients with non-alcoholic fatty liver disease. Nat. Commun. 5, 4309. 10.1038/ncomms5309 24978903 PMC4279183

[B35] Liu Q. Xue F. Meng J. Liu S.-S. Chen L.-Z. Gao H. (2019). TRIB1 rs17321515 and rs2954029 gene polymorphisms increase the risk of non-alcoholic fatty liver disease in Chinese Han population. Lipids Health Dis. 18, 61. 10.1186/s12944-019-1001-z 30851741 PMC6408849

[B36] Mahdessian H. Taxiarchis A. Popov S. Silveira A. Franco-Cereceda A. Hamsten A. (2014). TM6SF2 is a regulator of liver fat metabolism influencing triglyceride secretion and hepatic lipid droplet content. Proc. Natl. Acad. Sci. 111, 8913–8918. 10.1073/pnas.1323785111 24927523 PMC4066487

[B37] Mahfood Haddad T. Hamdeh S. Kanmanthareddy A. Alla V. M. (2017). Nonalcoholic fatty liver disease and the risk of clinical cardiovascular events: A systematic review and meta-analysis. Diabetes Metab. Syndr. Clin. Res. Rev. 11, S209–S216. 10.1016/j.dsx.2016.12.033 28017631

[B38] Mancina R. M. Dongiovanni P. Petta S. Pingitore P. Meroni M. Rametta R. (2016). The MBOAT7-TMC4 variant rs641738 increases risk of nonalcoholic fatty liver disease in individuals of European descent. Gastroenterology 150, 1219–1230.e6. 10.1053/j.gastro.2016.01.032 26850495 PMC4844071

[B39] Morrison A. E. Zaccardi F. Khunti K. Davies M. J. (2019). Causality between non-alcoholic fatty liver disease and risk of cardiovascular disease and type 2 diabetes: a meta-analysis with bias analysis. Liver Int. 39, 557–567. 10.1111/liv.13994 30358050

[B40] Nischalke H. D. Lutz P. Krämer B. Söhne J. Müller T. Rosendahl J. (2014). A common polymorphism in the NCAN gene is associated with hepatocellular carcinoma in alcoholic liver disease. J. Hepatol. 61, 1073–1079. 10.1016/j.jhep.2014.06.006 24946282

[B41] Onuma H. Tabara Y. Kawamoto R. Shimizu I. Kawamura R. Takata Y. (2010). The GCKR rs780094 polymorphism is associated with susceptibility of type 2 diabetes, reduced fasting plasma glucose levels, increased triglycerides levels and lower HOMA-IR in Japanese population. J. Hum. Genet. 55, 600–604. 10.1038/jhg.2010.75 20574426

[B42] Patil R. Sood G. K. (2017). Non-alcoholic fatty liver disease and cardiovascular risk. World J. Gastrointest. Pathophysiol. 8, 51–58. 10.4291/wjgp.v8.i2.51 28573067 PMC5437502

[B43] Petta S. Valenti L. Marchesini G. Marco V. D. Licata A. Cammà C. (2013). PNPLA3 GG Genotype and Carotid Atherosclerosis in Patients with Non-Alcoholic Fatty Liver Disease. PloS One 8, e74089. 10.1371/journal.pone.0074089 24069270 PMC3775795

[B44] Petta S. Miele L. Bugianesi E. Cammà C. Rosso C. Boccia S. (2014). Glucokinase regulatory protein gene polymorphism affects liver fibrosis in non-alcoholic fatty liver disease. PloS One 9, e87523. 10.1371/journal.pone.0087523 24498332 PMC3911959

[B45] Pingitore P. Pirazzi C. Mancina R. M. Motta B. M. Indiveri C. Pujia A. (2014). Recombinant PNPLA3 protein shows triglyceride hydrolase activity and its I148M mutation results in loss of function. Biochim. Biophys. Acta BBA Mol. Cell Biol. Lipids 1841, 574–580. 10.1016/j.bbalip.2013.12.006 24369119

[B46] Pingitore P. Dongiovanni P. Motta B. M. Meroni M. Lepore S. M. Mancina R. M. (2016). PNPLA3 overexpression results in reduction of proteins predisposing to fibrosis. Hum. Mol. Genet. 25, 5212–5222. 10.1093/hmg/ddw341 27742777 PMC5886043

[B47] Pirazzi C. Valenti L. Motta B. M. Pingitore P. Hedfalk K. Mancina R. M. (2014). PNPLA3 has retinyl-palmitate lipase activity in human hepatic stellate cells. Hum. Mol. Genet. 23, 4077–4085. 10.1093/hmg/ddu121 24670599 PMC4082369

[B48] Pirola C. J. Sookoian S. (2015). The dual and opposite role of the *TM6SF2* -rs58542926 variant in protecting against cardiovascular disease and conferring risk for nonalcoholic fatty liver: a meta-analysis: Hepatology, Month 2015. Hepatology 62, 1742–1756. 10.1002/hep.28142 26331730

[B49] Posadas-Sánchez R. López-Uribe Á. R. Posadas-Romero C. Pérez-Hernández N. Rodríguez-Pérez J. M. Ocampo-Arcos W. A. (2017). Association of the I148M/PNPLA3 (rs738409) polymorphism with premature coronary artery disease, fatty liver, and insulin resistance in type 2 diabetic patients and healthy controls. The GEA study. Immunobiology 222, 960–966. 10.1016/j.imbio.2016.08.008 27615511

[B50] Rüschenbaum S. Schwarzkopf K. Friedrich-Rust M. Seeger F. Schoelzel F. Martinez Y. (2018). Patatin-like phospholipase domain containing 3 variants differentially impact metabolic traits in individuals at high risk for cardiovascular events. Hepatol. Commun. 2, 798–806. 10.1002/hep4.1183 30027138 PMC6049070

[B51] Rafiq N. Bai C. Fang Y. Srishord M. McCullough A. Gramlich T. (2009). Long-term follow-up of patients with nonalcoholic fatty liver. Clin. Gastroenterol. Hepatol. 7, 234–238. 10.1016/j.cgh.2008.11.005 19049831

[B52] Rauch U. Feng K. Zhou X.-H. (2001). Neurocan: a brain chondroitin sulfate proteoglycan. Cell. Mol. Life Sci. CMLS 58, 1842–1856. 10.1007/PL00000822 11766883 PMC11337305

[B53] Rodríguez M. L. Silva L. F. Vangipurapu J. Modi S. Kuusisto J. Kaikkonen M. U. (2018). Functional variant in the GCKR gene affects lactate levels differentially in the fasting state and during hyperglycemia. Sci. Rep. 8, 1–8. 10.1038/s41598-018-34501-9 30375486 PMC6207693

[B54] Romeo S. Kozlitina J. Xing C. Pertsemlidis A. Cox D. Pennacchio L. A. (2008). Genetic variation in PNPLA3 confers susceptibility to nonalcoholic fatty liver disease. Nat. Genet. 40, 1461–1465. 10.1038/ng.257 18820647 PMC2597056

[B55] Rotman Y. Koh C. Zmuda J. M. Kleiner D. E. Liang T. J. (2010). The association of genetic variability in patatin-like phospholipase domain-containing protein 3 (PNPLA3) with histological severity of nonalcoholic fatty liver disease. Hepatology 52, 894–903. 10.1002/hep.23759 20684021 PMC2932770

[B56] Söderberg C. Stål P. Askling J. Glaumann H. Lindberg G. Marmur J. (2010). Decreased survival of subjects with elevated liver function tests during a 28-year follow-up. Hepatology 51, 595–602. 10.1002/hep.23314 20014114

[B57] Schunkert H. König I. R. Kathiresan S. Reilly M. P. Assimes T. L. Holm H. (2011). Large-scale association analysis identifies 13 new susceptibility loci for coronary artery disease. Nat. Genet. 43, 333–338. 10.1038/ng.784 21378990 PMC3119261

[B58] Simons N. Isaacs A. Koek G. H. Kuč S. Schaper N. C. Brouwers M. C. G. J. (2017). PNPLA3, TM6SF2, and MBOAT7 genotypes and coronary artery isease. Gastroenterology 152, 912–913. 10.1053/j.gastro.2016.12.020 28157516

[B59] Singal A. G. Manjunath H. Yopp A. C. Beg M. S. Marrero J. A. Gopal P., (2014). The effect of PNPLA3 on fibrosis progression and development of hepatocellular carcinoma: a meta-analysis. Am. J. Gastroenterol. 109, 325–334. 10.1038/ajg.2013.476 24445574 PMC5610907

[B60] Song J. Costa K. A. Fischer L. M. Kohlmeier M. Kwock L. Wang S. (2005). Polymorphism of the PEMT gene and susceptibility to nonalcoholic fatty liver disease (NAFLD). FASEB J. Off. Publ. Fed. Am. Soc Exp. Biol. 19, 1266–1271. 10.1096/fj.04-3580com PMC1256033 16051693

[B61] Sookoian S. Castaño G. O. Burgueño A. L. Gianotti T. F. Rosselli M. S. Pirola C. J. (2009). A nonsynonymous gene variant in the adiponutrin gene is associated with nonalcoholic fatty liver disease severity. J. Lipid Res. 50, 2111–2116. 10.1194/jlr.P900013-JLR200 19738004 PMC2739750

[B62] Sparsø T. Andersen G. Nielsen T. Burgdorf K. S. Gjesing A. P. Nielsen A. L. (2008). The GCKR rs780094 polymorphism is associated with elevated fasting serum triacylglycerol, reduced fasting and OGTT-related insulinaemia, and reduced risk of type 2 diabetes. Diabetologia 51, 70–75. 10.1007/s00125-007-0865-z 18008060

[B63] Speliotes E. K. Yerges-Armstrong L. M. Wu J. Hernaez R. Kim L. J. Palmer C. D. (2011). Genome-wide association analysis identifies variants associated with nonalcoholic fatty liver disease that have distinct effects on metabolic traits. PloS Genet. 7, e1001324. 10.1371/journal.pgen.1001324 21423719 PMC3053321

[B64] Stender S. Smagris E. Lauridsen B. K. Kofoed K. F. Nordestgaard B. G. Tybjærg-Hansen A. (2018). Relationship between genetic variation at PPP1R3B and levels of liver glycogen and triglyceride. Hepatology 67, 2182–2195. 10.1002/hep.29751 29266543 PMC5991995

[B65] Stepanova M. Younossi Z. M. (2012). Independent association between nonalcoholic fatty liver disease and cardiovascular disease in the US population. Clin. Gastroenterol. Hepatol. 10, 646–650. 10.1016/j.cgh.2011.12.039 22245962

[B66] Stewart J. Manmathan G. Wilkinson P. (2017). Primary prevention of cardiovascular disease: a review of contemporary guidance and literature. JRSM Cardiovasc. Dis. 6. 10.1177/2048004016687211 PMC5331469 28286646

[B67] Tam V. Patel N. Turcotte M. Bossé Y. Paré G. Meyre D. (2019). Benefits and limitations of genome-wide association studies. Nat. Rev. Genet. 20, 467–484. 10.1038/s41576-019-0127-1 31068683

[B68] Tan H. M. Mohamed R. B. Mohamed Z. Zain S. M. (2016). Phosphatidylethanolamine N-methyltransferase gene rs7946 polymorphism plays a role in risk of nonalcoholic fatty liver disease: evidence from meta-analysis. Pharmacogenet. Genomics 26, 88–95. 10.1097/FPC.0000000000000193 26636496

[B69] Tang C. S. Zhang H. Cheung C. Y. Y. Xu M. Ho J. C. Y. Zhou W. (2015). Exome-wide association analysis reveals novel coding sequence variants associated with lipid traits in Chinese. Nat. Commun. 6, 1–9. 10.1038/ncomms10206 PMC4703860 26690388

[B70] Targher G. Byrne C. D. Lonardo A. Zoppini G. Barbui C. (2016). Non-alcoholic fatty liver disease and risk of incident cardiovascular disease: A meta-analysis. J. Hepatol. 65, 589–600. 10.1016/j.jhep.2016.05.013 27212244

[B71] Valenti L. Al-Serri A. Daly A. K. Galmozzi E. Rametta R. Dongiovanni P. (2010). Homozygosity for the patatin-like phospholipase-3/adiponutrin I148M polymorphism influences liver fibrosis in patients with nonalcoholic fatty liver disease. Hepatol. Baltim. 51, 1209–1217. 10.1002/hep.23622 20373368

[B72] Vance D. E. (2013). Physiological roles of phosphatidylethanolamine N-methyltransferase. Biochim. Biophys. Acta BBA Mol. Cell Biol. Lipids 1831, 626–632. 10.1016/j.bbalip.2012.07.017 22877991

[B73] Varbo Anette B. M. Tybjærg-Hansen A. Grande P. Nordestgaard Børge G. (2011). TRIB1 and GCKR Polymorphisms, lipid levels, and risk of ischemic heart disease in the general population. Arterioscler. Thromb. Vasc. Biol. 31, 451–457. 10.1161/ATVBAHA.110.216333 21071687

[B74] Wang X. Liu Z. Wang K. Wang Z. Sun X. Zhong L. (2016). Additive effects of the risk alleles of PNPLA3 and TM6SF2 on non-alcoholic fatty liver disease (NAFLD) in a Chinese population. Front. Genet. 7, 140. 10.3389/fgene.2016.00140 27532011 PMC4969299

[B75] White D. L. Kanwal F. El-Serag H. B. (2012). Non-Alcoholic Fatty Liver Disease and Hepatocellular Cancer: A Systematic Review. Clin. Gastroenterol. Hepatol. Off. Clin. Pract. J. Am. Gastroenterol. Assoc. 10, 1342–1359.e2. 10.1016/j.cgh.2012.10.001 PMC3501546 23041539

[B76] Wispe’ J. R. Clark J. C. Burhans M. S. Kropp K. E. Korfhagen T. R. Whitsett J. A. (1989). Synthesis and processing of the precursor for human mangano-superoxide dismutase. Biochim. Biophys. Acta BBA Protein Struct. Mol. Enzymol. 994, 30–36. 10.1016/0167-4838(89)90058-7 2462451

[B77] Wu S. Wu F. Ding Y. Hou J. Bi J. Zhang Z. (2016). Association of non-alcoholic fatty liver disease with major adverse cardiovascular events: A systematic review and meta-analysis. Sci. Rep. 6, 33386. 10.1038/srep33386 27633274 PMC5026028

[B78] Xu L. Jiang C. Q. Lam T. H. Zhang W. S. Zhu F. Jin Y. L. (2017). Mendelian randomization estimates of alanine aminotransferase with cardiovascular disease: Guangzhou Biobank Cohort study. Hum. Mol. Genet. 26, 430–437. 10.1093/hmg/ddw396 28007909

[B79] Younossi Z. M. Koenig A. B. Abdelatif D. Fazel Y. Henry L. Wymer M. (2016). Global epidemiology of nonalcoholic fatty liver disease-Meta-analytic assessment of prevalence, incidence, and outcomes. Hepatol. Baltim. 64, 73–84. 10.1002/hep.28431 26707365

[B80] Younossi Z. M. Stepanova M. Rafiq N. Henry L. Loomba R. Makhlouf H. (2017). Nonalcoholic steatofibrosis independently predicts mortality in nonalcoholic fatty liver disease. Hepatol. Commun. 1, 421–428. 10.1002/hep4.1054 29404470 PMC5721410

[B81] Younossi Z. M. (2019). Non-alcoholic fatty liver disease – A global public health perspective. J. Hepatol. 70, 531–544. 10.1016/j.jhep.2018.10.033 30414863

[B82] Zain S. M. Mohamed Z. Mohamed R. (2015). Common variant in the glucokinase regulatory gene rs780094 and risk of nonalcoholic fatty liver disease: a meta-analysis. J. Gastroenterol. Hepatol. 30, 21–27. 10.1111/jgh.12714 25167786

[B83] Zeb I. Li D. Budoff M. J. Katz R. Lloyd-Jones D. Agatston A. (2016). Nonalcoholic fatty liver disease and incident cardiac events: the multi-ethnic study of atherosclerosis. J. Am. Coll. Cardiol. 67, 1965–1966. 10.1016/j.jacc.2016.01.070 27102512

[B84] Zhang Q.-H. Yin R.-X. Chen W.-X. Cao X.-L. Wu J.-Z. (2019). TRIB1 and TRPS1 variants, G × G and G × E interactions on serum lipid levels, the risk of coronary heart disease and ischemic stroke. Sci. Rep. 9, 2376. 10.1038/s41598-019-38765-7 30787327 PMC6382757

[B85] Zheng W. Wang L. Su X. Hu X.-F. (2014). MTP –493G > T Polymorphism and susceptibility to nonalcoholic fatty liver disease: a meta-analysis. DNA Cell Biol. 33, 361–369. 10.1089/dna.2013.2238 24588800

